# Inhibition of the BET family of epigenetic reader proteins: A novel principle for modulating gene expression in IgE‐activated mast cells

**DOI:** 10.1002/iid3.150

**Published:** 2017-03-13

**Authors:** Gianni Garcia‐Faroldi, Elin Rönnberg, Mirjana Grujic, Gunnar Pejler

**Affiliations:** ^1^ Department of Medical Biochemistry and MicrobiologyUppsala UniversityUppsalaSweden; ^2^ Department of Anatomy, Physiology and BiochemistrySwedish University of Agricultural SciencesUppsalaSweden

**Keywords:** BET proteins, epigenetics, IgE, IL‐6, mast cells

## Abstract

**Introduction:**

The BET family of bromodomain‐containing proteins constitute epigenetic readers that bind to acetylated lysine residues of core histones, thereby translating epigenetic histone marks to effects on gene expression. BET inhibitors are currently emerging as promising therapeutic agents for treatment of various pathological conditions. Here, we explored the potential of using BET inhibition to modulate IgE‐mediated responses in mast cells.

**Methods:**

We assessed the effects of BET inhibitors PFI‐1, I‐BET151, and I‐BET762 on responses downstream of mast cell activation through IgE receptor cross‐linking.

**Results:**

BET inhibitors were neither toxic for mast cells (at doses up to 20 μM), nor did they prevent IgE‐mediated mast cell degranulation. However, we found that BET inhibition, in particular by I‐BET151, suppressed IL‐6 gene expression and IL‐6 protein release in response to IgE‐mediated mast cell activation. This was observed in both bone marrow‐derived mast cells (BMMCs) and in mature peritoneal‐cell derived mast cells. Further analysis showed that BET inhibition also suppressed the expression of a number of additional genes of those that were upregulated by IgE receptor cross‐linking, including IL‐3, IL‐7R, CCR1, and ADAMTS9. However, BET inhibition was selective, i.e., several genes that were upregulated by IgE receptor cross‐linking were not affected by BET inhibitors.

**Conclusions:**

These findings suggest that BET inhibition can interfere with the upregulated expression of selected genes in mast cells activated by IgE receptor cross‐linking. Further, our findings introduce the concept of utilizing epigenetic mechanisms for modulating mast cell function in the context of IgE‐driven disease.

## Introduction

Mast cells are well known for their detrimental impact on allergic disease, but are also important players in a range of additional pathologies 1. The classical mode of mast cell activation, most notably in the context of allergic reactions, is through binding of multivalent antigen to IgE molecules bound to the high affinity IgE receptor, FcϵRI, on the mast cell surface. The ensuing IgE receptor cross‐linking will lead to extensive release of preformed mediators from granular stores, that is degranulation, but IgE‐mediated activation also results in the synthesis of numerous lipid‐derived pro‐inflammatory mediators and expression of a panel of genes coding for pro‐inflammatory compounds 2, 3. These latter include genes coding for cytokines, chemokines, and various growth factors 3. Altogether, mast cell activation through IgE receptor cross‐linking will thus lead to the release of a wide array of pro‐inflammatory compounds, which collectively gives rise to a powerful inflammatory reaction.

Based on the powerful impact of mast cells on allergic and other pathological conditions, there is a great demand for therapeutic strategies that can counteract harmful mast cell activities. Such strategies include the use of various mast cell stabilizers and anti‐IgE therapy to prevent mast cell degranulation, as well as antagonists to the individual mediators that are released following mast cell degranulation 4. However, there is also need for strategies that can interfere with gene expression patterns that are induced upon IgE‐mediated mast cell activation.

Histone modification by various mechanisms, including lysine acetylation, methylation, phosphorylation, and sumoylation is now established as a major epigenetic mechanism with wide consequences for gene expression 5, 6. The Bromodomain and extraterminal domain (BET) family of bromodomain proteins bind specifically to acetylated lysine residues in core histones, thereby transmitting the signal imposed by histone acetylation into effects on gene expression 7. Based on this principle, inhibitors of BET proteins have been developed to provide a novel means of directly modulating effects on gene expression. Such inhibitors have subsequently been evaluated in diverse settings and are currently regarded as promising therapeutic agents in various pathological conditions, including cancer, sepsis, and autoimmunity 8, 9, 10, 11, 12. However, the possibility of using BET inhibitors for modulating mast cell‐mediated events has hitherto not been explored.

Here, we investigated the effect of BET inhibition on mast cell function. Our findings identify BET inhibition as a novel means of modulating the expression of selected genes in response to IgE‐mediated mast cell activation. Hence, the use of BET inhibitors could represent a new strategy to directly interfere with gene expression patterns in pathological settings where mast cells are involved.

## Methods

### Reagents

Penicillin‐streptomycin, L‐glutamine, DMEM plus GlutaMAX, and minimum essential medium (MEM) non‐essential amino acids were from Invitrogen (Stockholm, Sweden), whereas Dulbecco Modified Essential Medium (DMEM), all salts for buffers, and the anti‐DNP IgE antibody were from Sigma (Stockholm, Sweden). DNP‐HSA was from Biosearch Technologies (Petaluma, CA). BET protein inhibitors PFI‐1 (PF‐6405761), I‐BET151 (GSK1210151A), and I‐BET762 (GSK525762) were from Sigma.

### Cell culture

Bone marrow‐derived mast cells (BMMCs) were obtained by culturing bone marrow cells from the femur and tibia of C57BL/6 mice as previously described 13. The animal experiments were approved by the local ethical committee (Uppsala djurförsöksetiska nämnd; approval no C 31/14). Peritoneal cell‐derived mast cells (PCMCs) 14 were obtained by maturation of cells from peritoneal lavage of C57BL/6 mice as described 13.

### Mast cell activation

Mast cells (BMMCs or PCMCs, at 1 × 10^6^ cells/ml) were sensitized overnight with IgE anti‐DNP at 0.1 μg/ml. The next day, cells were washed twice and suspended in fresh media or Tyrode's buffer (130 mM NaCl, 5 mM KCl, 1.4 mM CaCl_2_, 1 mM MgCl_2_, 5.6 mM glucose, 10 mM HEPES, and 0.1% BSA, pH 7.4) and stimulated for 1–24 h with DNP‐HSA (0.5 μg/ml). In samples where BET inhibitors were evaluated, mast cells were incubated for 1 h with BET inhibitor (PFI‐1, I‐BET151, or I‐BET762) at various concentrations, followed by activation with DNP‐HSA. For cytokine/chemokine release and Western blot analysis, activation with DNP‐HSA was performed for 4 or 24 h, while for mRNA expression, mast cells were activated for 1 h.

### Cytotoxicity test

Mast cells (BMMCs or PCMCs, at 10^6^ cells/ml) were incubated with serial dilutions of BET inhibitors (ranging from 10 nM to 20 μM) for 1 h. Cell viability was measured using the Cell Titer‐Blue® cell viability assay (Promega‐Invitrogen, Carlsbad, CA) according to the recommendations of the manufacturer.

### Cytokine/chemokine release

Supernatants from activated mast cells were analyzed for levels of IL‐6 and TNFα using ELISA kits as detailed by the manufacturer (eBiosciences, San Diego, CA).

### Beta‐hexosaminidase assay

Supernatants from activated mast cells were analyzed for their content of β‐hexosaminidase activity as described 15 as a measure of mast cell degranulation.

### Total RNA isolation, quantitative real time RT‐PCR, and gene array analysis

Total RNA isolation and quantitative real time RT‐PCR (qPCR) was performed as described 13. Hypoxanthine guanine phosphoribosyl transferase (Hprt) was used as housekeeping gene 13. Primers for IL‐3, −15 and 7r, CSF1, CCR1, and NLRP3 are described in Table 1. Primers for Nr4a3 [16], ADAMTS9 13, IL‐6 and −13 17, and Cpa3 18 were as described. Data were calculated as ΔΔC_t_ relative to HPRT, where values of the IgE‐sensitized sample were set as 1. In samples, where no signal corresponding to the sensitized controls (IgE only) were detected, values for the IgE + DNP group were set as 1. Gene array analysis was performed using Affymetrix GeneChip® expression arrays (GeneChip® Mouse Gene 1.0 ST Array), as described previously 19.

**Table 1:** Primers used for qPCR

Target	Forward primer	Reverse primer
IL‐3	ACCCACCGTTTAACCAGAACG	TGGACAGGTTTACTCTCCGAA
IL‐15	CATCCATCTCGTGCTACTTGTGTT	CATCTATCCAGTTGGCCTCTGTTT
IL‐7r	CCTTCGAAACTCCAGAACCCA	GACTAGGCCATACGACAGGTT
CSF1	AGTCTTGCTGACTGTTGGGG	TGTCTGTCCTCATCCTGGGT
CCR1	AGGCCCAGAAACAAAGTCTG	AGCAGTCTTTTGGCATGGAGT
NLRP3	CGAGACCTCTGGGAAAAAGCT	GCATACCATAGAGGAATGTGATGTACA

### Statistical analysis

Data shown are means ± standard error of the mean (SEM). Statistical analyses were performed by using GraphPad Prism 7.0 (GraphPad Software) and paired Student's *t‐*test for two‐tailed distributions. Differences were considered significant if the *P*‐values were 0.05 or less.

## Results

### BET inhibitors are non‐cytotoxic for mast cells

**Figure 1:**
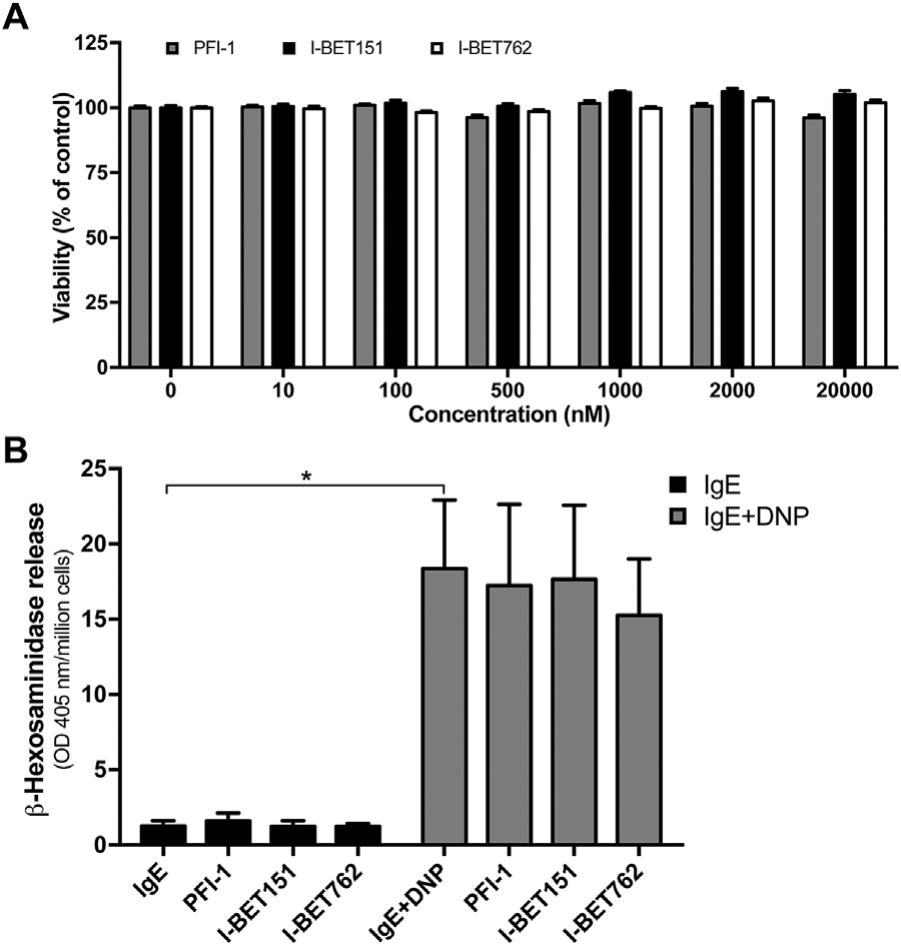
Effect of BET inhibition on viability and degranulation in bone marrow‐derived mast cells (BMMCs). (A) BMMCs (10^6 ^cells/ml) were treated for 1 h with the indicated concentrations of PFI‐1, I‐BET151, or I‐BET762. Cytotoxicity was evaluated using the Cell Titer‐Blue® cell viability assay. (B) BMMCs (10^6 ^cells/ml) were incubated for 1 h with 250 nM of PFI‐1, I‐BET151, or I‐BET762. Next, degranulation upon IgE‐receptor cross‐linking was measured after 1 h as release of β‐hexosaminidase. Data shown represent means ± SEM of three independent experiments. Paired Student's *t*‐test for two‐tailed distributions. *P < 0.05.

To evaluate the impact of BET inhibition on mast cell functionality, we first assessed whether BET inhibitors are cytotoxic to mast cells (BMMCs). At doses up to 20 μM, neither of three tested BET inhibitors (PFI‐1, I‐BET151, I‐BET762) were cytotoxic to mast cells after 1 h of incubation (Fig. 1A), and only limited cytotoxicity at concentrations above 50 nM were seen after 24 h of incubation (not shown). For the continuation of the study, BET inhibitors were used at concentrations and incubation times that were non‐toxic to mast cells (250 nM; 1 h).

### BET inhibition does not affect IgE‐mediated mast cell degranulation

Next, we assessed whether BET inhibition can interfere with mast cell degranulation in response to IgE receptor cross‐linking. To this end, mast cells were pre‐loaded with anti‐DNP IgE overnight, followed by IgE cross‐linking by addition of DNP‐HSA, either in the presence or absence of BET inhibitors. As seen in Figure 1B, IgE receptor cross‐linking resulted in a robust degranulation as measured by β‐hexosaminidase release. However, neither of the BET inhibitors used caused any detectable reduction of this response. Hence, BET inhibition does not affect IgE‐mediated mast cell degranulation.

### BET inhibition suppresses IL‐6 expression and release in response to IgE receptor cross‐linking

**Figure 2:**
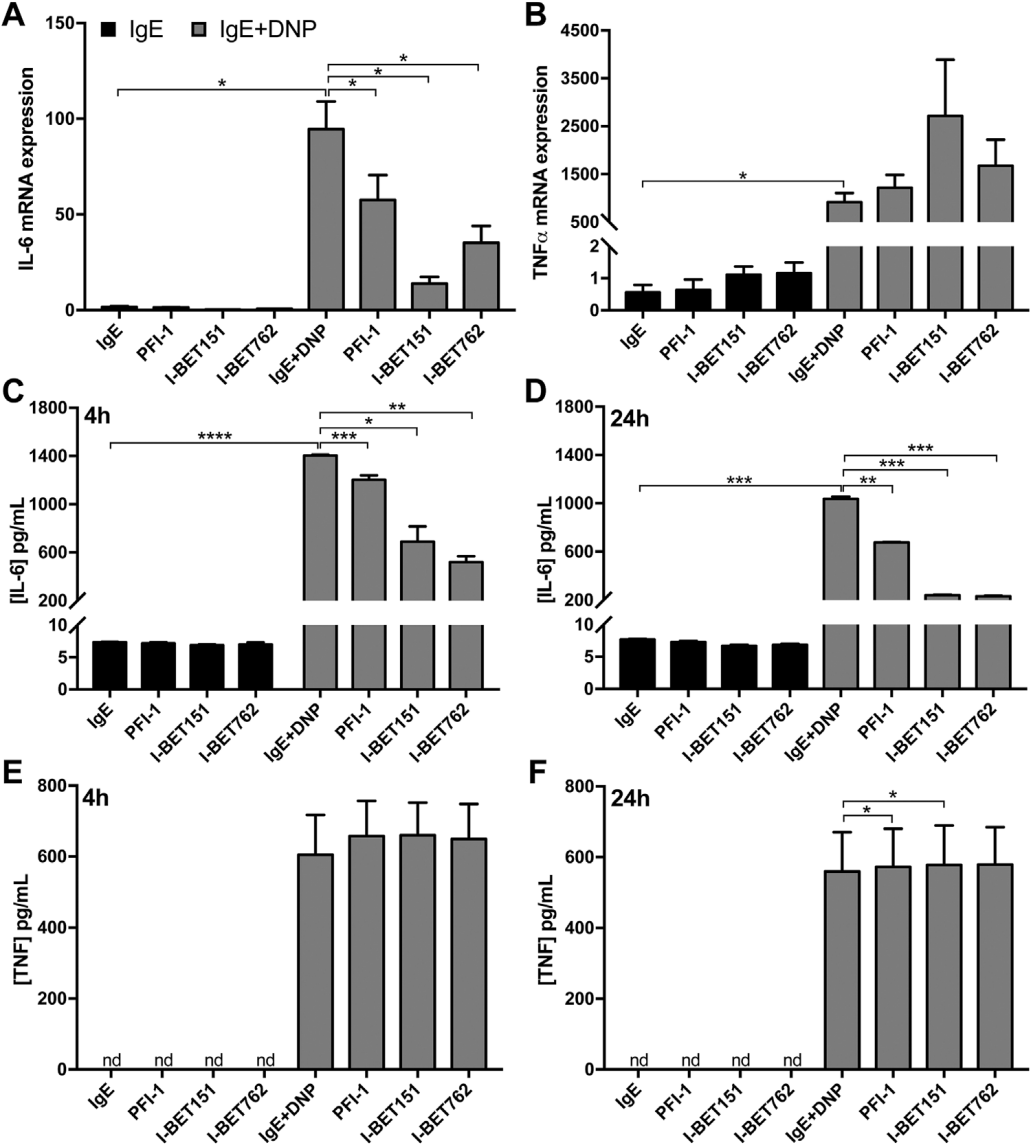
BET inhibitors suppress IL‐6 upregulation and release in activated bone marrow‐derived mast cells (BMMCs). BMMCs (10^6 ^cells/ml) were incubated for 1 h with 250 nM of PFI‐1, I‐BET151, or I‐BET762. Next, cells were activated by IgE receptor cross‐linking. After 1 h, cells were recovered for qPCR analysis of the expression of IL‐6 (A) and TNFα (B) mRNA. Culture supernatants were collected after 4 or 24 h as indicated, and the content of IL‐6 (C, D) and TNFα (E, F) in the supernatants was measured using ELISA. Data shown represent means ± SEM of three independent experiments. Paired Student's *t*‐test for two‐tailed distributions. *P < 0.05; **P < 0.01; ***P < 0.001; ****P < 0.0001.

Earlier reports have shown that BET inhibition can suppress IL‐6 expression in response to TLR4 ligation in macrophages 8, 20. In order to evaluate if BET inhibitors can modulate gene expression in IgE‐activated mast cells, we therefore first focused on IL‐6. As depicted in Figure 2A, IgE receptor cross‐linking caused a strong upregulation of IL‐6 gene expression. Moreover, it was seen that BET inhibition resulted in a profound reduction of this response. In particular, I‐BET151 proved to be a highly potent inhibitor of IL‐6 expression. In contrast, although TNFα gene expression was profoundly upregulated in response to IgE receptor cross‐linking in mast cells, neither of the BET inhibitors was capable of suppressing the TNFα induction (Fig. 2B). In agreement with these data, ELISA measurements showed that BET inhibition also suppressed the release of IL‐6 protein in response to IgE receptor cross‐linking (Fig. 2C and D), whereas no effect on the release of TNFα was seen (Fig. 2E and F).

### BET inhibition modulates IL‐6 expression in peritoneal cell‐derived mast cells

**Figure 3:**
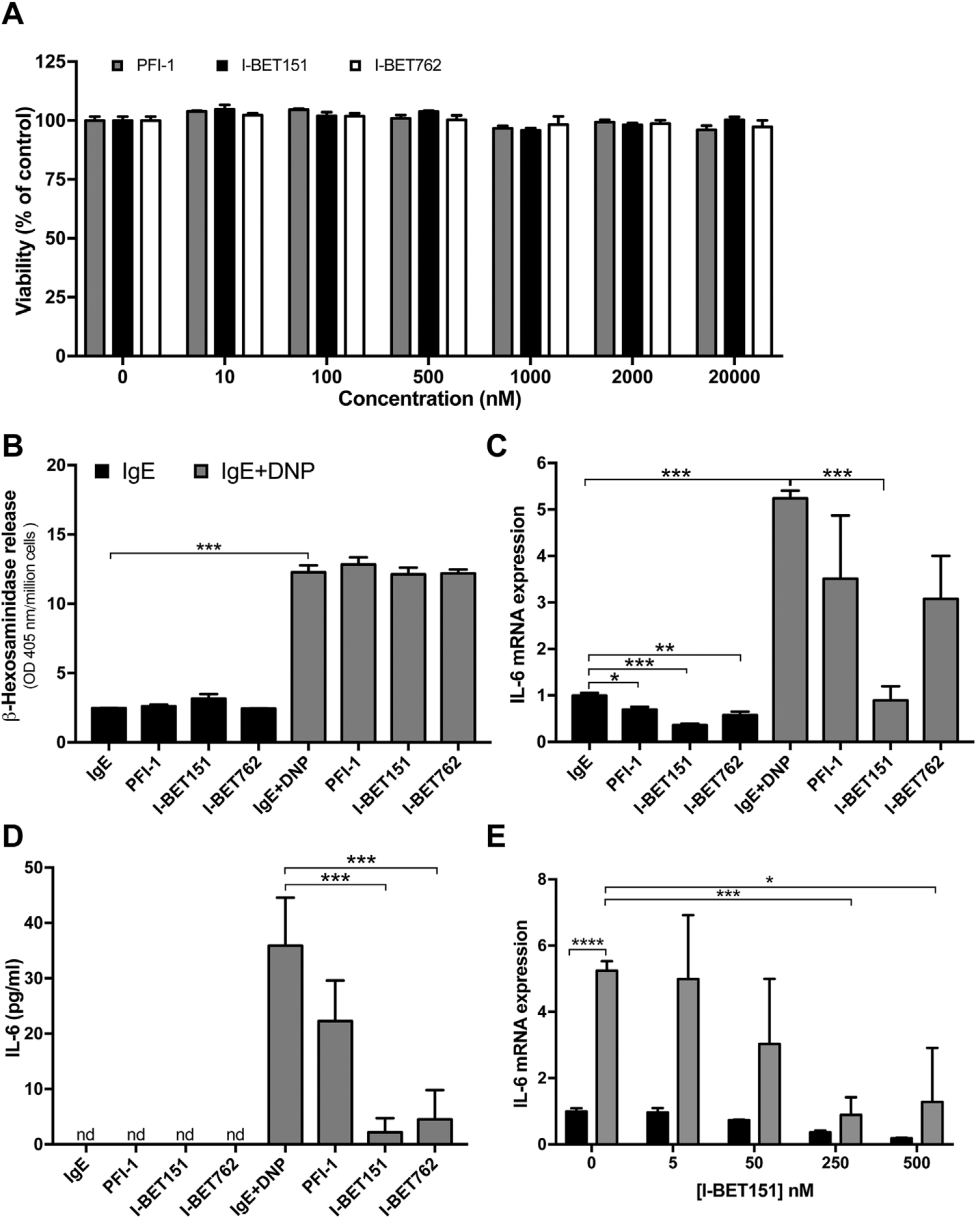
Effect of BET inhibition on peritoneal cell‐derived mast cells (PCMCs). (A) PCMCs (10^5 ^cells/ml) were treated for 1 h with indicated concentrations of PFI‐1, I‐BET151, or I‐BET762 and cytotoxicity was evaluated using the Cell Titer‐Blue® cell viability assay. (B) PCMCs (10^6 ^cells/ml) were incubated for 1 h with 250 nM of PFI‐1, I‐BET151, or I‐BET762. One hour after IgE receptor cross‐linking, the extent of degranulation was measured as release of β‐hexosaminidase. (C and D) PCMCs (10^6^cells/ml) were incubated for 1 h with 250 nM of PFI‐1, I‐BET151, or I‐BET762. Cells were then subjected to IgE receptor cross‐linking, followed by assessment of IL‐6 mRNA expression by qPCR (C; 1 h after activation) or measurement of IL‐6 release using ELISA (D; 24 h after activation). (E) PCMCs (10^6^ cells/ml) were incubated for 1 h with different concentrations of I‐BET151 followed by IgE receptor cross‐linking and quantification of IL‐6 mRNA expression using qPCR. Data shown represent means ± SEM of three independent experiments. Paired Student's *t*‐test for two‐tailed distributions. *P < 0.05; **P < 0.01; ***P < 0.001; ****P < 0.0001.

Although BMMCs are commonly used for studies of mast cell function, they are less mature than those mast cells that are found in vivo. To test the impact of BET inhibition on fully mature mast cells, we therefore developed PCMCs 14. Importantly, PCMCs have previously been shown to represent fully differentiated mast cells of connective tissue subtype 14. As for BMMCs, neither of the BET inhibitors at any of the tested concentrations showed cytotoxicity for PCMCs, when incubated for 1 h with the cells (Fig. 3A). Also after prolonged incubation (24 h), undetectable or only marginal cytotoxicity was seen at concentrations above 100 nM (data not shown). In agreement with the effects seen on BMMCs, BET inhibition did not affect the degranulation of PCMCs in response to IgE receptor cross‐linking (Fig. 3B). In further accordance with the effects on BMMCs, BET inhibition suppressed the expression of IL‐6 mRNA in response to IgE receptor cross‐linking and inhibited the release of IL‐6 protein in PCMCs (Fig. 3C and D). It was also seen that BET inhibition down‐regulated the baseline expression of IL‐6. As for BMMCs, BET inhibitor I‐BET151 was more effective than PFI‐1 and I‐BET762. A dose response experiment showed that optimal inhibition of IL‐6 gene expression was obtained at ∼250 nM of I‐BET151 (Fig. 3E).

### BET inhibition represses the expression of selected genes that are upregulated by IgE receptor cross‐linking

**Figure 4:**
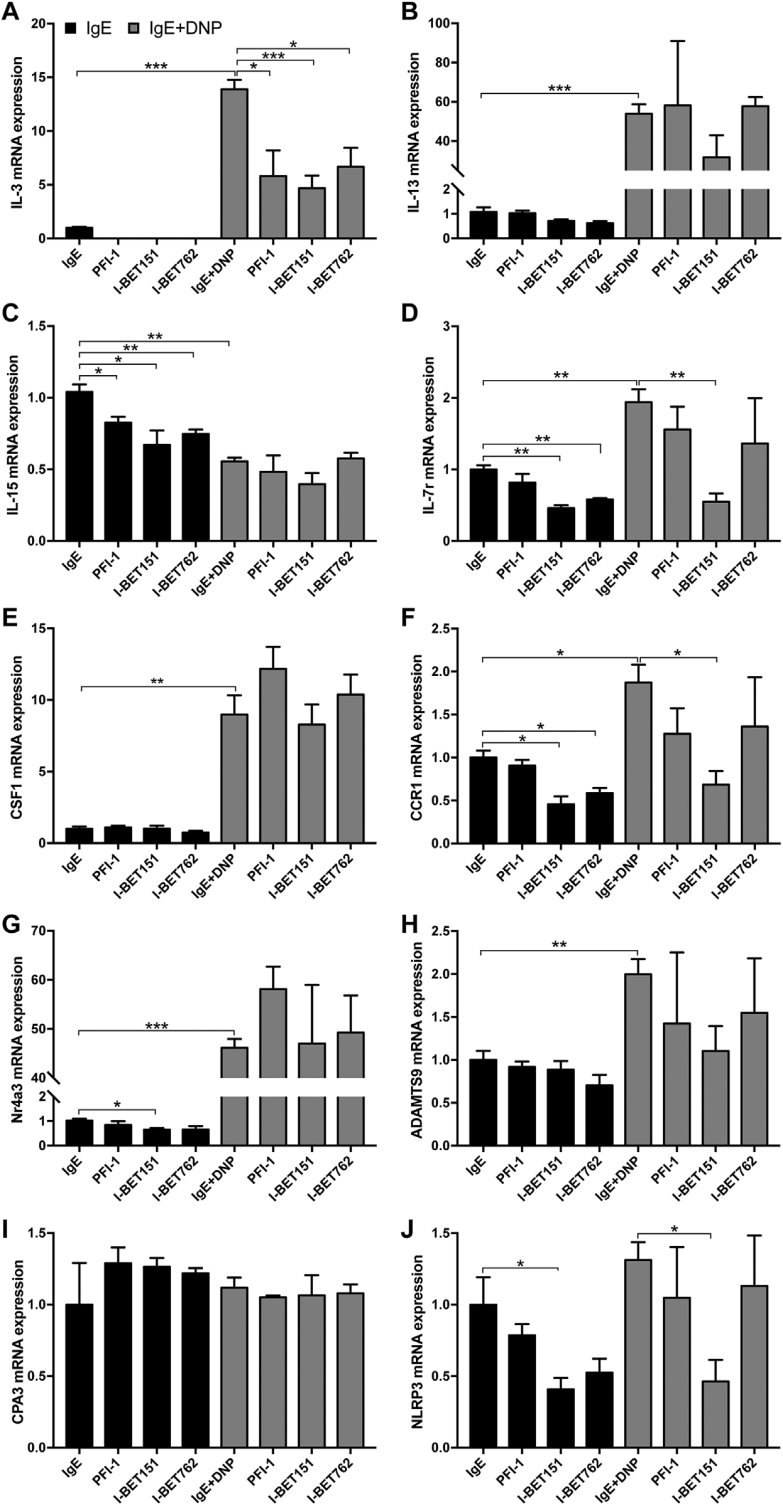
BET inhibition prevents upregulation of IL‐3, IL‐7r, CCR1, and NLRP3 in activated peritoneal cell‐derived mast cells (PCMCs). PCMCs (10^6^ cells/ml) were treated for 1 h with 250 nM of BET inhibitor and were then activated for 1 h by IgE‐receptor cross‐linking. mRNA levels corresponding to IL‐3 (A), IL‐13 (B), IL‐15 (C), IL‐7r (D), CSF1 (E), CCR1 (F), Nr4a3 (G), ADAMTS9 (H), CPA3 (I), and NLRP3 (J) were evaluated using qPCR. Data shown are means ± SEM of three independent experiments. Paired Student's *t*‐test for two‐tailed distributions. *P < 0.05; **P < 0.01; ***P < 0.001.

To provide a deeper insight into whether BET inhibition affects gene expression in IgE‐activated mast cells beyond the effects on IL‐6 (see Figs. 1 and 2), we performed a gene array analysis to search for additional genes affected by BET inhibitors. To this end, we performed a gene array analysis of PCMCs activated by IgE receptor cross‐linking, either in the absence or presence of BET inhibitor I‐BET151. As expected, this analysis suggested that a large number of protein‐encoding genes were upregulated in response to IgE receptor cross‐linking (Suppl. Table S1). Further, the gene array data indicated that only selected genes were substantially affected by BET inhibition (Suppl. Table S2). These included IL‐6, NLRP3, IL‐7R, IL‐3, CCR1, ADAMTS9, IL‐15, and IL‐13. To confirm the findings obtained through the gene array screen, we used quantitative real time PCR (qPCR). As shown in Figure 4, the qPCR analysis indeed revealed a robust induction of the IL‐3 (Fig. 4A), IL‐13 (Fig. 4B), IL‐7R Fig. 4D), CSF1 (Fig. 4E), CCR1 (Fig. 4F), and ADAMTS9 (Fig. 4H) genes in PCMCs subjected to IgE receptor cross‐linking. We also noted that Nr4a3, a gene that previously was shown to be one of the most highly induced genes in mast cells activated by various means 16, was strongly upregulated in IgE‐activated PCMCs (Fig. 4G). Out of these genes, the expression of IL‐3, IL7R (coding for the IL‐7 receptor), and CCR1 (receptor for chemokines CCL3, −5, −7, and −23) was suppressed by BET inhibition. Also the expression of NLRP3 (a key inflammasome component 21) was suppressed by BET inhibition, although the qPCR analysis did not support upregulated NLRP3 expression after IgE receptor cross‐linking (Fig. 4J). As for IL‐6 (see Fig. 3), BET inhibitor I‐BET151 was more effective than the other BET inhibitors. Notably, although the qPCR analysis confirmed an upregulated expression of IL‐13, CSF1, and Nr4a3 after IgE receptor cross‐linking, no effect of BET inhibition was seen on the expression of any of these genes. For ADAMTS9 (a metalloprotease), a trend of suppression was seen but this did not reach statistical significance. Further, BET inhibition did not affect the expression of the IL‐15 gene (Fig. 4B). We also tested the effect of BET inhibition on Cpa3, a secretory granule‐contained protease that serves as a major mast cell marker 2. Cpa3 expression was not affected by IgE receptor cross‐linking and, moreover, there was no effect of BET inhibition on Cpa3 expression (Fig. 4I).

**Figure 5:**
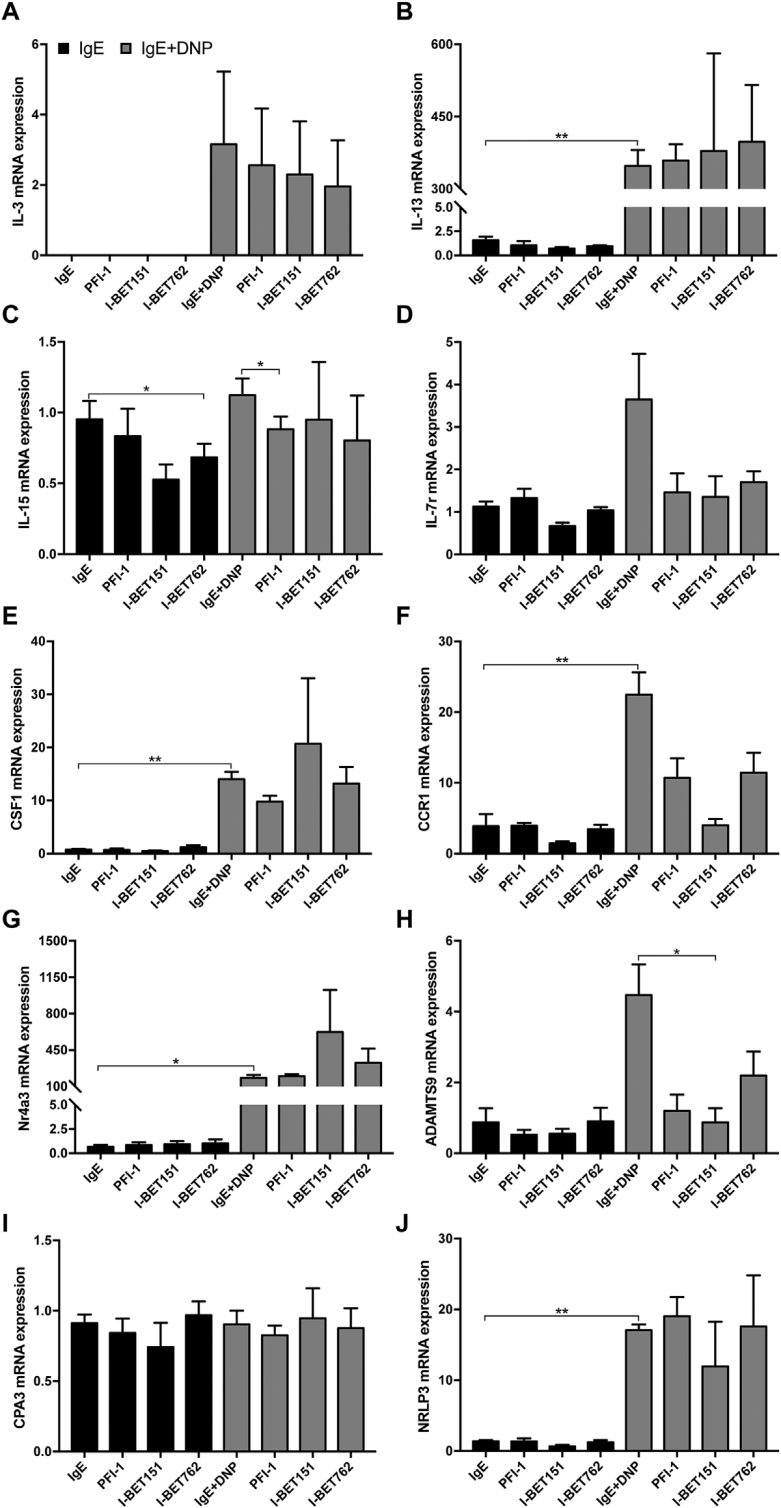
BET inhibition regulates gene expression in bone marrow‐derived mast cells (BMMCs). BMMCs (10^6^ cells/ml) were treated for 1 h with 250 nM of BET inhibitor and were then activated for 1 h by IgE‐receptor cross‐linking. mRNA levels corresponding to IL‐3 (A), IL‐13 (B), IL‐15 (C), IL‐7r (D), CSF1 (E), CCR1 (F), Nr4a3 (G), ADAMTS9 (H), CPA3 (I), and NLRP3 (J) were evaluated using qPCR. Data shown represent means ± SEM of three independent experiments. Paired Student's *t*‐test for two‐tailed distributions. *P < 0.05; **P < 0.01.

To extend these observations, we additionally evaluated whether BET inhibition had an impact on the expression of the corresponding genes in BMMCs, that is in less mature mast cells. As seen in Fig. 5, IgE receptor cross‐linking caused upregulation of the genes coding for IL‐3 (Fig. 5A), IL‐13 (Fig. 5B), CSF1 (Fig. 5E), CCR1 (Fig. 5F), Nr4a3 (Fig. 5G), ADAMTS9 (Fig. 5H), and NLRP3 (Fig. 5J) along with a trend of upregulated expression of the Il‐7R gene (Fig. 5D), that is similar findings as for PCMCs. However, BMMCs were generally less responsive to BET inhibition in comparison with PCMCs, with only the expression of the ADAMTS9 (Fig. 5H) and IL‐15 (Fig. 5C) genes being significantly repressed by any of the BET inhibitors. However, trends of inhibition were seen for the IL‐7R (Fig. 5D) and CCR1 (Fig. 5F) genes. As in PCMCs, Cpa3 expression was neither affected by IgE receptor cross‐linking, nor by BET inhibition (Fig. 5I).

## Discussion

BET inhibitors have previously been assessed in a variety of settings and are currently emerging as promising therapeutic agents (see Introduction). However, the possibility of using this type of inhibitors to intervene with mast cell functions has, to our knowledge, not been addressed before. Here, we show that gene expression in mast cells activated by IgE receptor cross‐linking can be modulated by inhibition of the BET proteins. One important implication of these findings is that responses downstream of IgE‐mediated mast cell activation can be influenced by epigenetic mechanisms. In line with our findings, it was recently shown that interference with BRD4, one of the BET family members can suppress growth and induce apoptosis in neoplastic mast cells 22. Hence, modulation of BET proteins could potentially represent a novel means of modulating mast cell‐mediated events in a variety of settings.

It is apparent from the present study that BET inhibitors do not interfere with mast cell degranulation. It is therefore unlikely that BET inhibition has effects on the immediate response downstream of mast cell activation, that is events related to effects of the preformed mast cell mediators released from secretory granules. Instead, BET inhibition was shown to modulate gene expression patterns in response to IgE receptor cross‐linking, thus implicating BET inhibitors as possible agents to modulate events occurring in the late phase responses following mast cell activation. However, it was noted that BET inhibition also suppressed the baseline expression of several of the assessed genes. Hence, BET‐mediated regulation of gene expression in mast cells is not restricted to situations in which they are activated.

One of the main findings was that BET inhibition potently suppressed the upregulation of IL‐6, both at the gene and protein level. This is thus in analogy with previous studies demonstrating that BET inhibitors can suppress IL‐6 expression in macrophages stimulated with TLR4 ligands 8, 20. Based on the findings presented here, we may propose that BET inhibition can be used as a therapeutic strategy to modulate IL‐6 gene expression in mast cells. Importantly, mast cell‐derived IL‐6 has been implicated in a variety of pathological contexts, including atherosclerosis 23, stroke pathology 24, arthritis 25, Th17 skewing 26, mastocytosis 27, and diabetes 28. Hence, modulation of IL‐6 expression by BET inhibition could have a therapeutic potential in such settings.

Besides the IL‐6 gene, we here identified a number of additional genes whose expression in IgE‐activated mast cells was affected by BET inhibition. These included IL‐3, IL‐7R, and CCR1, all of which have established and important functions in inflammatory responses. Of note, IL‐3 is a potent growth factor for mast cells 29, and the suppression of IL‐3 expression by BET inhibition might thus interfere with IL‐3‐driven mast cell proliferation. Potentially, reduction of mast cell numbers by inhibition of BET proteins could thus have beneficial effects under circumstances when mast cells account for aggravating activities, for example in allergic asthma and mastocytosis. We also found that BET inhibition suppressed the expression of NLRP3. It is thus possible that BET inhibition could represent a novel strategy for interfering with inflammasome activity. In addition, the expression of ADAMTS9, a protease that recently was identified as a mast cell signature gene 30 and to be highly upregulated by IgE receptor cross‐linking 13, was suppressed in mast cells subjected to BET inhibition. As ADAMTS9 is implicated in connective tissue remodeling events 31, BET inhibition could thus counteract such processes orchestrated by mast cells.

In summary, this study identifies BET inhibitors as novel agents with capacity to modulate mast cell gene expression. Importantly, the use of BET inhibition to modulate gene expression in mast cells constitutes a novel principle for intervening with mast cell function, in which gene expression is directly targeted rather than being modulated by targeting of signaling molecules that act upstream of the gene transcription apparatus.

## Authors' Contributions

GGF planned and performed experiments, interpreted data, and wrote parts of the manuscript; ER planned and performed experiments, interpreted data, and contributed to the writing of the manuscript; MG planned and performed experiments, interpreted data; GP conceived of the study, planned experiments, interpreted data, and wrote the manuscript.

## Conflict of Interest

The authors do not have any conflicts of interest in relation to this study.

## Supporting information

Additional supporting information may be found in the online version of this article at the publisher's web‐site

**Table S1**. Genes upregulated in peritoneal cell‐derived mast cells activated by IgE receptor crosslinking.

**Table S2**. Genes downregulated by BET inhibitor 151 in peritoneal cell‐derived mast cells activated by IgE receptor crosslinking.

## References

[1] Voehringer, D. 2013 Protective and pathological roles of mast cells and basophils. Nat. Rev. Immunol. 13:362–375. 23558889 10.1038/nri3427

[2] Wernersson, S. , and G. Pejler . 2014 Mast cell granules: armed for battle. Nat. Rev. Immunol. 14:478–494. 24903914 10.1038/nri3690

[3] Galli, S. J. , S. Nakae , and M. Tsai . 2005 Mast cells in the development of adaptive immune responses. Nat. Immunol. 6:135–142. 15662442 10.1038/ni1158

[4] Reber, L. L. , and N. Frossard . 2014 Targeting mast cells in inflammatory diseases. Pharmacol. Ther. 142:416–435. 24486828 10.1016/j.pharmthera.2014.01.004

[5] Kouzarides, T. 2007 Chromatin modifications and their function. Cell 128:693–705. 17320507 10.1016/j.cell.2007.02.005

[6] Strahl, B. D. , and C. D. Allis . 2000 The language of covalent histone modifications. Nature 403:41–45. 10638745 10.1038/47412

[7] Gallenkamp, D. , K. A. Gelato , B. Haendler , and H. Weinmann . 2014 Bromodomains and their pharmacological inhibitors. ChemMedChem 9:438–464. 24497428 10.1002/cmdc.201300434

[8] Nicodeme, E. , K. L. Jeffrey , U. Schaefer , S. Beinke , S. Dewell , C. W. Chung , R. Chandwani , I. Marazzi , P. Wilson , H. Coste , et al. 2010 Suppression of inflammation by a synthetic histone mimic. Nature 468:1119–1123. 21068722 10.1038/nature09589 PMC5415086

[9] Delmore, J. E. , G. C. Issa , M. E. Lemieux , P. B. Rahl , J. Shi , H. M. Jacobs , E. Kastritis , T. Gilpatrick , R. M. Paranal , J. Qi , et al. 2011 BET bromodomain inhibition as a therapeutic strategy to target c‐Myc. Cell 146:904–917. 21889194 10.1016/j.cell.2011.08.017 PMC3187920

[10] Dawson, M. A. , R. K. Prinjha , A. Dittmann , G. Giotopoulos , M. Bantscheff , W. I. Chan , S. C. Robson , C. W. Chung , C. Hopf , M. M. Savitski , et al. 2011 Inhibition of BET recruitment to chromatin as an effective treatment for MLL‐fusion leukaemia. Nature 478:529–533. 21964340 10.1038/nature10509 PMC3679520

[11] Bandukwala, H. S. , J. Gagnon , S. Togher , J. A. Greenbaum , E. D. Lamperti , N. J. Parr , A. M. Molesworth , N. Smithers , K. Lee , J. Witherington , et al. 2012 Selective inhibition of CD4+ T‐cell cytokine production and autoimmunity by BET protein and c‐Myc inhibitors. Proc. Natl. Acad. Sci. U.S.A. 109:14532–14537. 22912406 10.1073/pnas.1212264109 PMC3437860

[12] Mele, D. A. , A. Salmeron , S. Ghosh , H. R. Huang , B. M. Bryant , and J. M. Lora . 2013 BET bromodomain inhibition suppresses TH17‐mediated pathology. J. Exp. Med. 210:2181–2190. 24101376 10.1084/jem.20130376 PMC3804955

[13] Garcia‐Faroldi, G. , E. Rönnberg , A. Orro , G. Calounova , B. Guss , A. Lundequist , and G. Pejler . 2013 ADAMTS: novel proteases expressed by activated mast cells. Biol. Chem. 394:291–305. 23154421 10.1515/hsz-2012-0270

[14] Malbec, O. , K. Roget , C. Schiffer , B. Iannascoli , A. R. Dumas , M. Arock , and M. Daeron . 2007 Peritoneal cell‐derived mast cells: an in vitro model of mature serosal‐type mouse mast cells. J. Immunol. 178:6465–6475. 17475876 10.4049/jimmunol.178.10.6465

[15] Rönnberg, E. , and G. Pejler . 2012 Serglycin: the master of the mast cell. Methods Mol. Biol. 836:201–217. 22252637 10.1007/978-1-61779-498-8_14

[16] Lundequist, A. , G. Calounova , H. Wensman , E. Ronnberg , and G. Pejler . 2011 Differential regulation of Nr4a subfamily nuclear receptors following mast cell activation. Mol. Immunol. 48:1753–1761. 21621845 10.1016/j.molimm.2011.04.017

[17] Garcia‐Vilas, J. A. , M. A. Medina , F. R. Melo , G. Pejler , and G. Garcia‐Faroldi . 2015 Damnacanthal inhibits IgE receptor‐mediated activation of mast cells. Mol. Immunol. 65:86–93. 25656801 10.1016/j.molimm.2015.01.008

[18] Garcia‐Faroldi, G. , C. E. Rodriguez , J. L. Urdiales , J. M. Perez‐Pomares , J. C. Davila , G. Pejler , F. Sanchez‐Jimenez , and I. Fajardo . 2010 Polyamines are present in mast cell secretory granules and are important for granule homeostasis. PLoS ONE 5:e15071. 21151498 10.1371/journal.pone.0015071 PMC2994821

[19] Rönnberg, E. , B. Guss , and G. Pejler . 2010 Infection of mast cells with live streptococci causes a toll‐like receptor 2‐ and cell‐cell contact‐dependent cytokine and chemokine response. Infect. Immun. 78:854–864. 19933827 10.1128/IAI.01004-09 PMC2812202

[20] Barrett, E. , S. Brothers , C. Wahlestedt , and E. Beurel . 2014 I‐BET151 selectively regulates IL‐6 production. Biochim. Biophys. Acta. 1842:1549–1555. 24859008 10.1016/j.bbadis.2014.05.013 PMC4125491

[21] Haneklaus, M. , and L. A. O'Neill . 2015 NLRP3 at the interface of metabolism and inflammation. Immunol. Rev. 265:53–62. 25879283 10.1111/imr.12285

[22] Wedeh, G. , S. Cerny‐Reiterer , G. Eisenwort , H. Herrmann , K. Blatt , E. Hadzijusufovic , I. Sadovnik , L. Mullauer , J. Schwaab , T. Hoffmann , et al. 2015 Identification of bromodomain‐containing protein‐4 as a novel marker and epigenetic target in mast cell leukemia. Leukemia 29:2230–2237. 26055303 10.1038/leu.2015.138 PMC4610040

[23] Sun, J. , G. K. Sukhova , P. J. Wolters , M. Yang , S. Kitamoto , P. Libby , L. A. MacFarlane , J. Mallen‐St Clair , and G. P. Shi . 2007 Mast cells promote atherosclerosis by releasing proinflammatory cytokines. Nat. Med. 13:719–724. 17546038 10.1038/nm1601

[24] Arac, A. , M. A. Grimbaldeston , A. R. Nepomuceno , O. Olayiwola , M. P. Pereira , Y. Nishiyama , A. Tsykin , G. J. Goodall , U. Schlecht , H. Vogel , et al. 2014 Evidence that meningeal mast cells can worsen stroke pathology in mice. Am. J. Pathol. 184:2493–2504. 25134760 10.1016/j.ajpath.2014.06.003 PMC4188278

[25] van der Velden, D. , H. M. Lagraauw , A. Wezel , P. Launay , J. Kuiper , T. W. Huizinga , R. E. Toes , I. Bot , and J. N. Stoop . 2016 Mast cell depletion in the preclinical phase of collagen‐induced arthritis reduces clinical outcome by lowering the inflammatory cytokine profile. Arthritis Res. Ther. 18:138. 27296719 10.1186/s13075-016-1036-8 PMC4907027

[26] Ganeshan, K. , and P. J. Bryce . 2012 Regulatory T cells enhance mast cell production of IL‐6 via surface‐bound TGF‐beta. J. Immunol. 188:594–603. 22156492 10.4049/jimmunol.1102389 PMC3253181

[27] Desai, A. , M. Y. Jung , A. Olivera , A. M. Gilfillan , C. Prussin , A. S. Kirshenbaum , M. A. Beaven , and D. D. Metcalfe . 2016 IL‐6 promotes an increase in human mast cell numbers and reactivity through suppression of suppressor of cytokine signaling 3. J. Allergy. Clin. Immuno. 137:1863–1871. 10.1016/j.jaci.2015.09.059 PMC4899186 26774658

[28] Liu, J. , A. Divoux , J. Sun , J. Zhang , K. Clement , J. N. Glickman , G. K. Sukhova , P. J. Wolters , J. Du , C. Z. Gorgun , et al. 2009 Genetic deficiency and pharmacological stabilization of mast cells reduce diet‐induced obesity and diabetes in mice. Nat. Med. 15:940–945. 19633655 10.1038/nm.1994 PMC2736875

[29] Lantz, C. S. , J. Boesiger , C. H. Song , N. Mach , T. Kobayashi , R. C. Mulligan , Y. Nawa , G. Dranoff , and S. J. Galli . 1998 Role for interleukin‐3 in mast‐cell and basophil development and in immunity to parasites. Nature 392:90–93. 9510253 10.1038/32190

[30] Dwyer, D. F. , N. A. Barrett , and K. F. Austen . 2016 Immunological Genome Project C. Expression profiling of constitutive mast cells reveals a unique identity within the immune system. Nat. Immunol. 17:878–887. 27135604 10.1038/ni.3445 PMC5045264

[31] Lemarchant S , M. Pruvost , J. Montaner , E. Emery , D. Vivien , K. Kanninen , and J. Koistinaho . ADAMTS proteoglycanases in the physiological and pathological central nervous system. J. Neuroinflammation 2013 10:133. 24176075 10.1186/1742-2094-10-133 PMC4228433

