# Supplementary material for: Inhibition of the BET family of epigenetic reader proteins: A novel principle for modulating gene expression in IgE‐activated mast cells
Source: Immun Inflamm Dis. 2017 Mar 13;5(2):141–50. doi: 10.1002/iid3.150 (PMC5418135; doi:10.1002/iid3.150)
Supplement: Supplementary file 1 — Table S1. Genes upregulated in peritoneal cell‐derived mast cells activated by IgE receptor crosslinking. [file IID3-5-141-s001.docx]

**Suppl Table 1.** Genes upregulated in peritoneal cell-derived mast cells activated by IgE receptor crosslinking.

| **Gene name** | **Gene description** | **Log2-fold change: IgE/DNP vs. IgE** |
| --- | --- | --- |
| Olr1 | oxidized low density lipoprotein (lectin-like) receptor 1 | 6,60 |
| Nr4a3 | nuclear receptor subfamily 4, group A, member 3 | 6,42 |
| Ccl3 | chemokine (C-C motif) ligand 3 | 5,44 |
| Il13 | interleukin 13 | 4,63 |
| Nr4a1 | nuclear receptor subfamily 4, group A, member 1 | 4,26 |
| Nfkbid | nuclear factor of kappa light polypeptide gene enhancer in B cells inhibitor, delta | 3,89 |
| Gadd45b | growth arrest and DNA-damage-inducible 45 beta | 3,79 |
| Egr2 | early growth response 2 | 3,71 |
| Clec7a | C-type lectin domain family 7, member a | 3,38 |
| Tnfsf14 | tumor necrosis factor (ligand) superfamily, member 14 | 3,25 |
| Egr1 | early growth response 1 | 3,25 |
| Rcan1 | regulator of calcineurin 1 | 3,09 |
| Nr4a2 | nuclear receptor subfamily 4, group A, member 2 | 3,03 |
| Csf1 | colony stimulating factor 1 (macrophage) | 2,98 |
| Rabgef1 | RAB guanine nucleotide exchange factor (GEF) 1 | 2,34 |
| Hnrnpk | heterogeneous nuclear ribonucleoprotein K | 2,28 |
| Prrg4 | proline rich Gla (G-carboxyglutamic acid) 4 (transmembrane) | 2,26 |
| Cass4 | Cas scaffolding protein family member 4 | 2,23 |
| Fam110c | family with sequence similarity 110, member C | 2,19 |
| Atf3 | activating transcription factor 3 | 2,12 |
| Adora2a | adenosine A2a receptor | 2,12 |
| Ripk2 | receptor (TNFRSF)-interacting serine-threonine kinase 2 | 2,08 |
| Ccrn4l | CCR4 carbon catabolite repression 4-like (S. cerevisiae) | 2,08 |
| Hectd2 | HECT domain containing 2 | 2,07 |
| Fosl2 | fos-like antigen 2 | 2,07 |
| Ccl7 | chemokine (C-C motif) ligand 7 | 2,03 |
| Emp1 | epithelial membrane protein 1 | 1,95 |
| Nfkbiz | nuclear factor of kappa light polypeptide gene enhancer in B cells inhibitor, zeta | 1,95 |
| Ftl1 | ferritin light chain 1 | 1,94 |
| Stk38l | serine/threonine kinase 38 like | 1,92 |
| Actg1 | actin, gamma, cytoplasmic 1 | 1,92 |
| Zc3h12c | zinc finger CCCH type containing 12C | 1,89 |
| Cdc42 | cell division cycle 42 | 1,88 |
| Hsp90aa1 | heat shock protein 90, alpha (cytosolic), class A member 1 | 1,86 |
| Ehd1 | EH-domain containing 1 | 1,85 |
| Zswim4 | zinc finger SWIM-type containing 4 | 1,85 |
| Grhl1 | grainyhead-like 1 (Drosophila) | 1,84 |
| Frmd6 | FERM domain containing 6 | 1,83 |
| Bbs10 | Bardet-Biedl syndrome 10 (human) | 1,82 |
| Ptger4 | prostaglandin E receptor 4 (subtype EP4) | 1,79 |
| Mrgprx1 | MAS-related GPR, member X1 | 1,78 |
| Dnajb1 | DnaJ (Hsp40) homolog, subfamily B, member 1 | 1,77 |
| Srxn1 | sulfiredoxin 1 homolog (S. cerevisiae) | 1,71 |
| Cish | cytokine inducible SH2-containing protein | 1,71 |
| Tle1 | transducin-like enhancer of split 1, homolog of Drosophila E(spl) | 1,69 |
| Rhof | ras homolog gene family, member f | 1,68 |
| Slc37a2 | solute carrier family 37 (glycerol-3-phosphate transporter), member 2 | 1,66 |
| Ero1l | ERO1-like (S. cerevisiae) | 1,66 |
| Vim | vimentin | 1,65 |
| Rhbdf2 | rhomboid 5 homolog 2 (Drosophila) | 1,63 |
| Il7r | interleukin 7 receptor | 1,62 |
| Myl12a | myosin, light chain 12A, regulatory, non-sarcomeric | 1,61 |
| Slc7a1 | solute carrier family 7 (cationic amino acid transporter, y+ system), member 1 | 1,61 |
| Rrad | Ras-related associated with diabetes | 1,60 |
| Cited2 | Cbp/p300-interacting transactivator, with Glu/Asp-rich carboxy-terminal domain, 2 | 1,60 |
| Trim16 | tripartite motif-containing 16 | 1,58 |
| Arl5b | ADP-ribosylation factor-like 5B | 1,57 |
| Adamts9 | a disintegrin-like and metallopeptidase (reprolysin type) with thrombospondin type 1 motif, 9 | 1,56 |
| Spty2d1 | SPT2, Suppressor of Ty, domain containing 1 (S. cerevisiae) | 1,55 |
| Il3 | interleukin 3 | 1,54 |
| Il6 | interleukin 6 | 1,51 |
| Nedd9 | neural precursor cell expressed, developmentally down-regulated gene 9 | 1,47 |
| Hmox1 | heme oxygenase (decycling) 1 | 1,45 |
| Arhgap5 | Rho GTPase activating protein 5 | 1,45 |
| Vegfc | vascular endothelial growth factor C | 1,42 |
| Tnfrsf12a | tumor necrosis factor receptor superfamily, member 12a | 1,41 |
| Mfsd12 | major facilitator superfamily domain containing 12 | 1,40 |
| Ptgs2 | prostaglandin-endoperoxide synthase 2 | 1,38 |
| Sertad1 | SERTA domain containing 1 | 1,38 |
| Rpl39 | ribosomal protein L39 | 1,37 |
| Slfn2 | schlafen 2 | 1,37 |
| Havcr2 | hepatitis A virus cellular receptor 2 | 1,36 |
| Slain2 | SLAIN motif family, member 2 | 1,36 |
| Chka | choline kinase alpha | 1,35 |
| Rap1b | RAS related protein 1b | 1,35 |
| Exoc5 | exocyst complex component 5 | 1,35 |
| Loxl2 | lysyl oxidase-like 2 | 1,35 |
| Cyp51 | cytochrome P450, family 51 | 1,34 |
| Plaur | plasminogen activator, urokinase receptor | 1,34 |
| Map2k3 | mitogen-activated protein kinase kinase 3 | 1,34 |
| Cd69 | CD69 antigen | 1,31 |
| Cdk17 | cyclin-dependent kinase 17 | 1,30 |
| Gnb2l1 | guanine nucleotide binding protein (G protein), beta polypeptide 2 like 1 | 1,30 |
| Fam107b | family with sequence similarity 107, member B | 1,29 |
| Pim1 | proviral integration site 1 | 1,29 |
| n-R5s156 | nuclear encoded rRNA 5S 156 | 1,29 |
| Homer1 | homer homolog 1 (Drosophila) | 1,28 |
| Map3k8 | mitogen-activated protein kinase kinase kinase 8 | 1,28 |
| Klf10 | Kruppel-like factor 10 | 1,28 |
| Tagap | T cell activation Rho GTPase activating protein | 1,27 |
| Rilpl2 | Rab interacting lysosomal protein-like 2 | 1,27 |
| Spry2 | sprouty homolog 2 (Drosophila) | 1,27 |
| Tubb6 | tubulin, beta 6 class V | 1,26 |
| Nab2 | Ngfi-A binding protein 2 | 1,26 |
| Vmn1r20 | vomeronasal 1 receptor 20 | 1,24 |
| Nfkb2 | nuclear factor of kappa light polypeptide gene enhancer in B cells 2, p49/p100 | 1,23 |
| Snhg3 | small nucleolar RNA host gene (non-protein coding) 3 | 1,21 |
