# Supplementary material for: Inhibition of the BET family of epigenetic reader proteins: A novel principle for modulating gene expression in IgE‐activated mast cells
Source: Immun Inflamm Dis. 2017 Mar 13;5(2):141–50. doi: 10.1002/iid3.150 (PMC5418135; doi:10.1002/iid3.150)
Supplement: Supplementary file 2 — Table S2. Genes downregulated by BET inhibitor 151 in peritoneal cell‐derived mast cells activated by IgE receptor crosslinking. [file IID3-5-141-s002.docx]

**Suppl. Table 2.** Genes downregulated by BET inhibitor 151 in peritoneal cell-derived mast cells activated by IgE receptor crosslinking.

| **Gene** | **Gene description** | **Log2-fold change; IgE/DNP + I-BET151 vs. IgE/DNP** |
| --- | --- | --- |
| Il6 | interleukin 6 | -2,05 |
| Nlrp3 | NLR family, pyrin domain containing 3 | -1,91 |
| Mrgprx1 | MAS-related GPR, member X1 | -1,57 |
| Emp1 | epithelial membrane protein 1 | -1,41 |
| Il7r | interleukin 7 receptor | -1,31 |
| Mecom | MDS1 and EVI1 complex locus | -1,27 |
| Il3 | interleukin 3 | -1,17 |
| Gpr171 | G protein-coupled receptor 171 | -1,15 |
| Ccr1 | chemokine (C-C motif) receptor 1 | -1,13 |
| Adamts9 | a disintegrin-like and metallopeptidase (reprolysin type) with thrombospondin type 1 motif, 9 | -1,13 |
| Apol9b | apolipoprotein L 9b | -1,10 |
| Sp110 | Sp110 nuclear body protein | -1,08 |
| Il15 | interleukin 15 | -1,06 |
| Il13 | interleukin 13 | -1,03 |
| Vegfc | vascular endothelial growth factor C | -1,00 |
| Zfp608 | zinc finger protein 608 | -0,99 |
| Adora2a | adenosine A2a receptor | -0,95 |
| Havcr2 | hepatitis A virus cellular receptor 2 | -0,91 |
| Rmi1 | RMI1, RecQ mediated genome instability 1, homolog (S. cerevisiae) | -0,91 |
| Pbp2 | phosphatidylethanolamine binding protein 2 | -0,90 |
| Hnrnpk | heterogeneous nuclear ribonucleoprotein K | -0,90 |
| Frmd4b | FERM domain containing 4B | -0,90 |
| Zfp455 | zinc finger protein 455 | -0,89 |
| Olfr988 | olfactory receptor 988 | -0,89 |
| Adamts6 | a disintegrin-like and metallopeptidase (reprolysin type) with thrombospondin type 1 motif, 6 | -0,88 |
| Itk | IL2 inducible T cell kinase | -0,88 |
| Cdc42 | cell division cycle 42 | -0,87 |
| Adamts1 | a disintegrin-like and metallopeptidase (reprolysin type) with thrombospondin type 1 motif, 1 | -0,86 |
| Rrad | Ras-related associated with diabetes | -0,84 |
| Synj2 | synaptojanin 2 | -0,83 |
| Rnf180 | ring finger protein 180 | -0,83 |
| Ighv1-54 | immunoglobulin heavy variable V1-54 | -0,83 |
| Adamts5 | a disintegrin-like and metallopeptidase (reprolysin type) with thrombospondin type 1 motif, 5 (aggrecanase-2) | -0,80 |
| Sprr2b | small proline-rich protein 2B | -0,78 |
| Omp | olfactory marker protein | -0,76 |
| Clec4d | C-type lectin domain family 4, member d | -0,76 |
| Ptpn22 | protein tyrosine phosphatase, non-receptor type 22 (lymphoid) | -0,76 |
| Tlr13 | toll-like receptor 13 | -0,75 |
| Hgf | hepatocyte growth factor | -0,75 |
| Cass4 | Cas scaffolding protein family member 4 | -0,74 |
| Prrg4 | proline rich Gla (G-carboxyglutamic acid) 4 (transmembrane) | -0,73 |
| Fgf2 | fibroblast growth factor 2 | -0,72 |
| Gbp11 | guanylate binding protein 11 | -0,72 |
| Gpr183 | G protein-coupled receptor 183 | -0,71 |
| Trib2 | tribbles homolog 2 (Drosophila) | -0,71 |
| Vmn2r17 | vomeronasal 2, receptor 17 | -0,70 |
| Olfr1196 | olfactory receptor 1196 | -0,70 |
| Speer4e | spermatogenesis associated glutamate (E)-rich protein 4e | -0,70 |
| St8sia4 | ST8 alpha-N-acetyl-neuraminide alpha-2,8-sialyltransferase 4 | -0,69 |
| Tuba3b | tubulin, alpha 3B | -0,69 |
| Zfy1 | zinc finger protein 1, Y linked | -0,68 |
| n-R5s1 | nuclear encoded rRNA 5S 1 | -0,68 |
| Olfr99 | olfactory receptor 99 | -0,68 |
| C4b | complement component 4B (Chido blood group) | -0,67 |
| Gpr34 | G protein-coupled receptor 34 | -0,69 |
| Tnfsf8 | tumor necrosis factor (ligand) superfamily, member 8 | -0,67 |
